# The atomic force microscope as a mechano–electrochemical pen

**DOI:** 10.3762/bjnano.2.70

**Published:** 2011-10-04

**Authors:** Christian Obermair, Andreas Wagner, Thomas Schimmel

**Affiliations:** 1 Institute of Applied Physics and Center for Functional Nanostructures (CFN), South Campus, Karlsruhe Institute of Technology (KIT), 76128 Karlsruhe, Germany; 2 Institute of Nanotechnology, North Campus, Karlsruhe Institute of Technology (KIT), 76128 Karlsruhe, Germany

**Keywords:** atomic force microscopy, deposition, electrochemistry, nanoelectronics, nanofabrication, nanolithography, nanotechnology, NEMS and MEMS, scanning probe lithography

## Abstract

We demonstrate a method that allows the controlled writing of metallic patterns on the nanometer scale using the tip of an atomic force microscope (AFM) as a “mechano–electrochemical pen”. In contrast to previous experiments, no voltage is applied between the AFM tip and the sample surface. Instead, a passivated sample surface is activated locally due to lateral forces between the AFM tip and the sample surface. In this way, the area of tip–sample interaction is narrowly limited by the mechanical contact between tip and sample, and well-defined metallic patterns can be written reproducibly. Nanoscale structures and lines of copper were deposited, and the line widths ranged between 5 nm and 80 nm, depending on the deposition parameters. A procedure for the sequential writing of metallic nanostructures is introduced, based on the understanding of the passivation process. The mechanism of this mechano–electrochemical writing technique is investigated, and the processes of site-selective surface depassivation, deposition, dissolution and repassivation of electrochemically deposited nanoscale metallic islands are studied in detail.

## Introduction

The controlled, patterned, electrochemical deposition of metals at predefined positions on the nanometer scale is of great interest for numerous applications including in the fields of microelectronics, nanoscale electronics and nano-electromechanical systems (NEMS). Considerable progress was achieved recently in the field of self-organized electrochemical patterning of nanowires. In thin-film electrolytes, regular arrays of nanowires were grown in flat electrochemical cells at reduced temperatures. A dramatic increase of the mechanical yield strength of the nanowires of more than one order of magnitude as compared to bulk values was reported recently [1–3]. Significant progress was also achieved in the field of the controlled electrochemical deposition of metals for the fabrication of atomic-scale contacts and switches. By electrochemical deposition of nanoscale silver contacts and subsequent electrochemical cycling, an electrically controllable single-atom relay was demonstrated, which allows the controlled switching of an electrical current by the control-voltage-induced movement of just a single atom [4–7]. In this way, a single-atom transistor was demonstrated as a quantum electronic device operating reproducibly at room temperature. At the same time, the scanning tunneling microscope (STM) and the atomic force microscope (AFM) represent techniques that allow surface manipulation on the nanometer scale and even on the atomic scale [8–21]. As shown in Don Eigler’s pioneering work [8], the tip of an STM allows the assembling of structures on a surface, atom by atom. Early experiments demonstrated that the tip of an electrochemical STM can also be used for local electrochemical deposition. Material electrochemically deposited on an STM tip was subsequently transferred to the surface [22–23], allowing controlled metallic nanopatterning of surfaces. Improvements of STM-based techniques also include the use of elaborate voltage-pulse sequences [24–25].

While much work was performed using the STM as a tool for electrochemical patterning, only a few attempts exist utilizing the AFM as a tool for controlled site-selective electrochemical deposition of metals on surfaces. Initial experiments performed by LaGraff and Gewirth [26–27] demonstrated that the influence of the scanning tip can lead to both reduction or enhancement of copper deposition on the surface of copper single crystals. In further work we demonstrated the local electrochemical deposition of metal islands mechanically induced with the tip of an AFM [28]. Herein, we demonstrate that by combined passivation/depassivation of surfaces, complex metallic nanostructures can be selectively deposited by using the tip of an AFM as a mechano–electrochemical pen in the sense that it allows the local mechanical depassivation of a formerly passivated substrate surface for local electrochemical deposition.

## Results and Discussion

**Figure 1:**
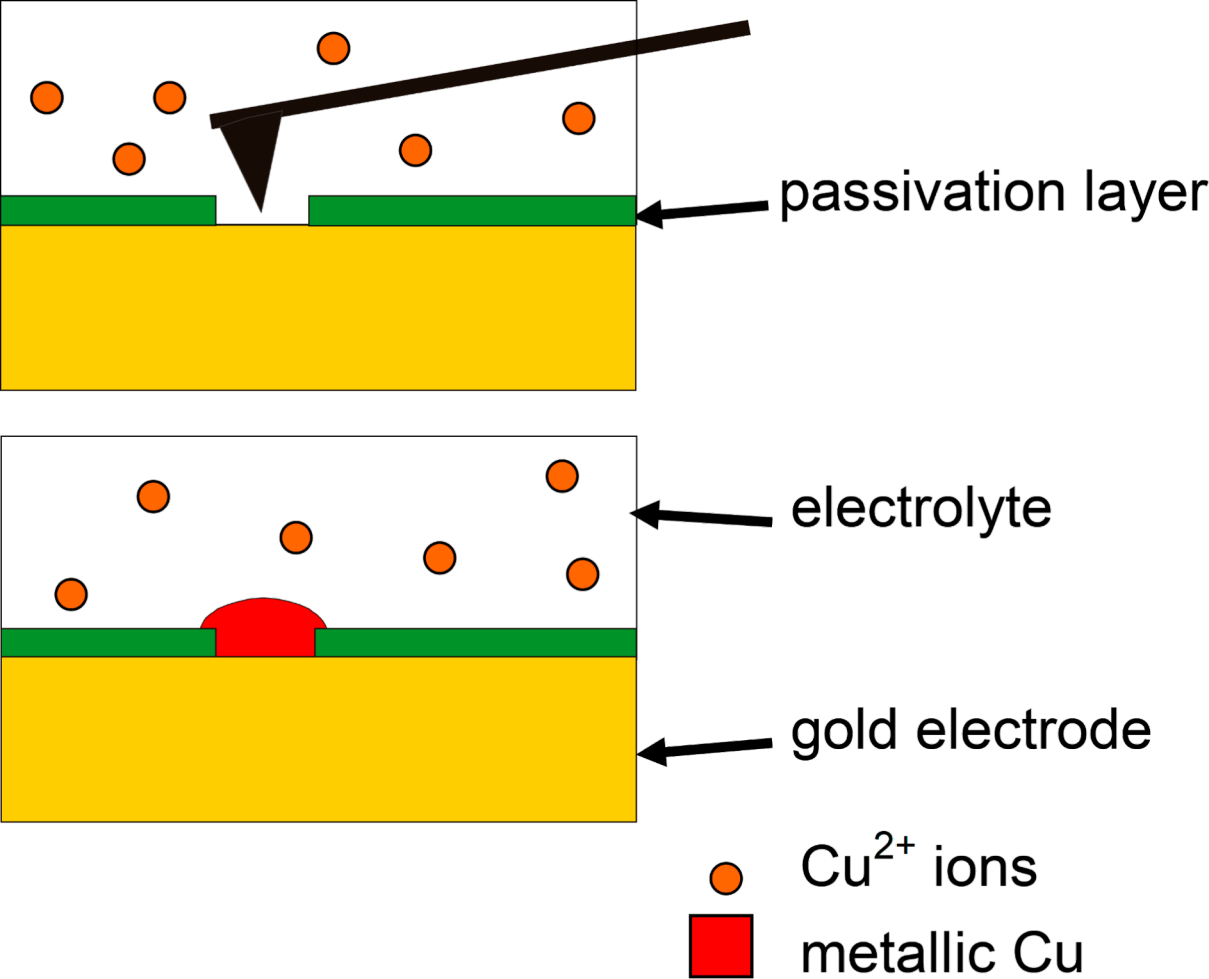
Schematic diagram of a gold electrode with a passivation layer, in an electrolyte containing Cu^2+^ ions. An electrochemical potential, appropriate for tip-induced electrochemical deposition (cf. text), was applied to the gold electrode. The passivation layer can be removed or reduced, site-selectively, with the tip of an AFM (top). In this way metallic copper is only deposited selectively at the areas of the gold substrate that are scanned by the AFM tip (bottom).

The basic principle of the structuring process applied in our experiments is illustrated in the schematic diagram in Figure 1. The gold substrate, which serves as the working electrode, is covered by a native passivation layer. This passivation layer consists of oxo-anions of the electrolyte, such as sulphate or hydrogen sulfate, which are well known from literature to cover metal films in their presence [29–33]. Alternatively, thiol molecules were used in our experiments as an organic passivation layer. These thiol molecules have a higher adhesion to the substrate but are not necessary for a precise deposition and are therefore not discussed in any more detail below.

While an electrochemical potential appropriate for tip-induced electrochemical deposition is applied to the gold electrode, simultaneously the passivation layer is partially removed, site-selectively, with the tip of an AFM. The appropriate electrochemical potential is determined by cyclic voltammetry, and a cathodic potential is selected so as to be too low to lead to an overall growth of the metal film in spite of the passivation layer. Thus, metal is *only* deposited at the areas of the gold substrate that were scanned by the AFM tip and where, therefore, the passivation layer inhibiting the electrochemical deposition is locally destroyed. The method described above is not limited to the deposition of single nanostructures activated with the tip of an AFM. We found that if the gold electrode is exposed to the electrolyte at a neutral potential for deposition (holding potential), the surface is again re-covered by a passivation layer, including coverage of the newly deposited structures. By this mechanism of self-passivation, previously deposited structures do not continue to grow when the deposition potential is applied again. Rather, we find that after a short time of approximately 10–20 s after stopping the deposition, the newly deposited structures are passivated. This means that if we deposit a metal nanostructure in the way described above and we wait for 10–20 s after finishing deposition, this structure will not grow further when we apply a deposition potential again after this 10–20 s pause. On the other hand, if we start scanning these structures again with the AFM tip at the forces mentioned above, on the order of 10 nN, electrochemical growth continues as long as the deposition potential is applied and the scanning continues. This provides important information concerning the mechanism of the passivation, depassivation and repassivation. It gives the time scale of the repassivation process, which for the electrochemical conditions given in our experiment is on the order of 10 s. The results also indicate that when scanning at tip–sample forces on the order of 10 nN, passivated surfaces of the deposited copper are again depassivated, and that repassivation is efficiently prohibited locally within the scanning area of the AFM tip.

**Figure 2:**
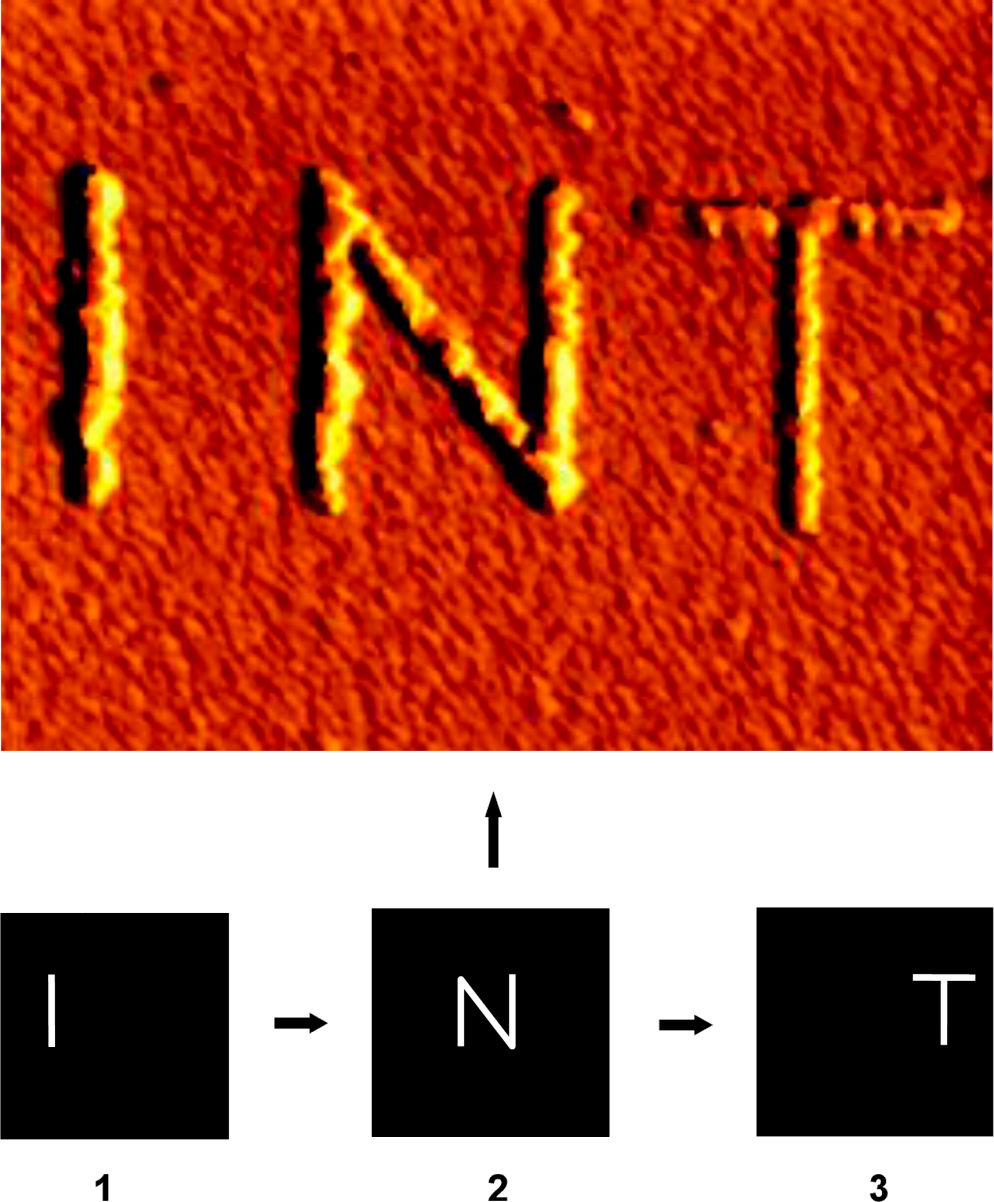
Sequential writing and passivation of Cu nanostructures. Top: AFM image of sequentially written, unconnected Cu nanostructures (“INT”), site-selectively deposited with the tip of the AFM. Scan size 1.8 µm × 1.8 µm. Bottom: Image sections (1–3) of the program used to control the shape of the separate structures (“I”, “N”, “T”) scanned by the AFM tip. The distinct structures were deposited by consecutive deposition in the indicated order (1–3), by applying a deposition potential of −85 mV vs Cu/Cu^2+^. Between the scans of the different structures an electrochemical holding potential of −35 mV vs Cu/Cu^2+^ was applied for about 10 s to allow for the repassivation of the previously deposited nanostructures.

At the same time these results open possibilities for the controlled *sequential* writing of several independent nanostructures on the same substrate chip: After one copper nanostructure has been deposited and after the electrodeposition as well as the AFM scanning process has subsequently been stopped, it is sufficient to wait for just 10–20 s. After this time, the surface of the copper appears to be passivated, and one can write a new structure neighboring the first one without inducing further growth of the previously written structure. To demonstrate this sequential deposition process for the example of three separate nanostructures, an appropriate electrochemical deposition potential of −85 mV for tip-induced deposition was applied to the gold electrode. Then the shape of the letter “I” was scanned 50 times with the AFM tip (tip speed: 8 µm/s). Subsequently a holding potential of −35 mV was applied for a period of 10 s. After that, the letters “N” and “T” were scanned in a similar way with a holding-potential period between the structuring cycles for each letter. Figure 2 (bottom) shows the image sections (1–3) of the program used to control the shape of the separate structures. Subsequent AFM imaging leads to the image in Figure 2 (top) (scan size 1.8 µm × 1.8 µm). Three unconnected Cu nanostructures (“INT”) were deposited successively and site-selectively based on the “activation” or de-passivation by the scanning tip of the AFM.

**Figure 3:**
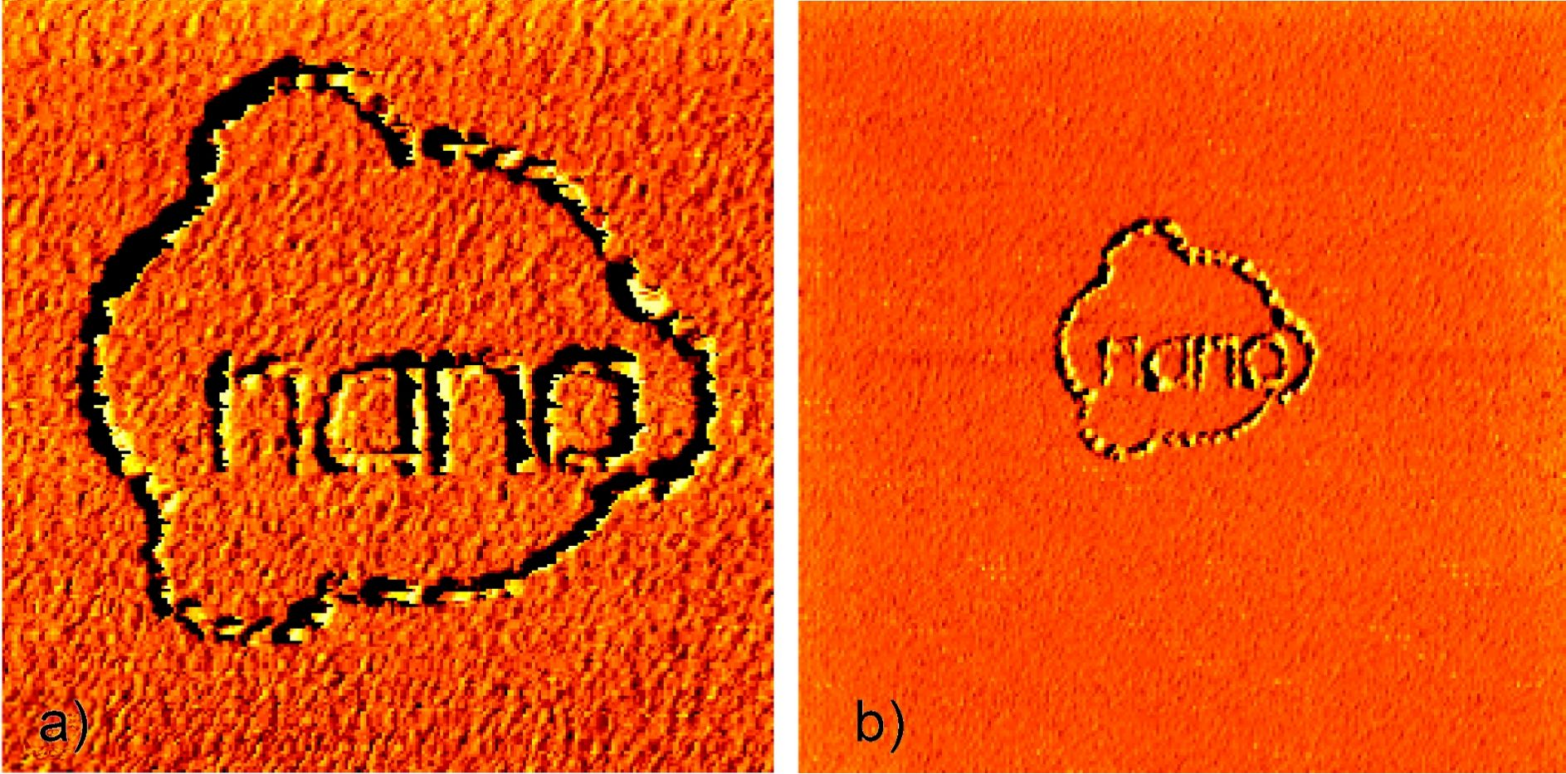
In situ AFM image demonstrating the selectivity of the tip-induced electrochemical copper deposition (“nano” + ring) from a Cu^2+^ electrolyte onto a polycrystalline gold substrate. a) Cu island structure deposited by sequential writing (deposition potential: −60 mV vs Cu/Cu^2+^). Between writing the separate structures an electrochemical holding potential of −35 mV vs Cu/Cu^2+^ was applied for a period of 10 s. b) AFM image of the same Cu nanostructure at larger scan size, demonstrating the selectivity of the tip-induced deposition process; that is, no Cu islands are found in the surface areas that were not activated by the AFM tip during deposition. Scan size: a) 2.5 µm × 2.5 µm, b) 6 µm × 6 µm.

A further example of this method is given in Figure 3a. Here an even more complex structure was produced following the same procedure. All the separate nanostructures have nearly the same height, indicating that during the holding-potential period the surface, including the previously deposited nanostructures, repassivates and does not continue growing during further deposition. The AFM image of Figure 3b shows the Cu nanostructure of Figure 3a at a larger scan size, demonstrating the selectivity of the tip-induced deposition process. The scan size is 2.5 µm × 2.5 µm in a) and 6 µm × 6 µm in b), respectively. Even at the larger scan size of Figure 3b, not one single Cu island is found outside the locations depassivated by the AFM tip during deposition. The above experiments demonstrate that electrodeposition can be induced locally with the tip of an AFM. As no potential was applied to the tip of the AFM and the experiments were reproduced both with insulating and with electrically conducting tips, the locally selective deposition is most likely related to the mechanical interaction between tip and sample during the scanning process. This is further supported by the observation that the mere presence of the tip in contact with the sample does not lead to locally selective deposition. The scanning process, i.e., the movement of the tip relative to the sample, is necessary to induce local electrodeposition.

Obviously, the scanning AFM tip mechanically activates deposition sites and/or nucleation centers for local copper deposition. A possible explanation for the observed phenomena is the assumed presence of a passivating layer on the gold surface that prevents deposition of copper on the gold surface at values of the overpotential between −60 mV and 0 V. Due to the mechanical interaction between the tip and sample, this passivating layer is disrupted locally. This would explain the selective local copper deposition along the lines were the tip was scanned. This explanation is also in agreement with the observation that lateral forces occurring between tip and sample during the scanning process can be used to induce rupture of chemical bonds mechanically [17–18]. As the experiments have been performed under environmental conditions, especially in the presence of oxygen, the formation of a surface layer on the gold substrate involving copper oxide/hydroxide and/or other compounds such as thiols is possible.

The alternative explanation that the tip induces defects within the gold surface itself, which, in turn, could possibly act as nucleation sites for the subsequent copper deposition, may well explain a somewhat enhanced copper deposition within the scanning area of the tip. Such a mechanism, however, cannot explain our experimental results for several reasons:

1. No damage was found on the gold surfaces after tip-induced deposition and subsequent dissolution of the deposited copper.
2. No memory effect is observed, when a further deposition experiment is performed on the same area of the gold surface after such a dissolution of the copper deposited in the first deposition experiment. This indicates that the gold surface is still intact and that there is no prepatterned sequence of nucleation centers due to defects induced during the first deposition experiment.
3. Furthermore, it is difficult to explain the high selectivity of the deposition process by merely assuming a substrate-defect-nucleated deposition mechanism. The substrate for deposition in our experiments is not a metal single crystal, but rather thermally evaporated polycrystalline gold, which was not annealed and which even before interaction with the AFM tip would exhibit a high density of defects (steps, kinks, dislocations, etc.). In our experiments, metal deposition was observed selectively only at the positions were the AFM tip was scanned.

Finally, tip-induced defects on the gold surface cannot explain the effects of repassivation and depassivation of the once-passivated copper structures described above.

## Conclusion

To conclude, we have reported the controlled and site-selective electrochemical deposition of metallic nanopatterns, which were induced with the tip of an atomic force microscope used as a “mechano–electrochemical pen”. The deposition led to the controlled writing of metallic patterns and lines, with line widths between 5 nm and 80 nm, depending on the structuring parameters. The process is highly selective, leading to electrochemical deposition only within the areas activated by the AFM tip. The mechanism can be explained as a mechanical depassivation of the substrate surface by the scanning tip, leading to local deposition in the depassivated area. If the tip is scanned repeatedly along a given line pattern while a deposition potential is applied, this will result in the site-selective deposition along the scanning path of the AFM tip. The results, which include the sequential writing and subsequent in situ repassivation of newly written structures, open perspectives for novel lithographic processes mechanically activated with the tip of an AFM.

## Experimental

**Electrochemical AFM setup:** We used a home-built AFM with a tube scanner and a beam-deflection detection system with a four-quadrant photodetector, allowing the simultaneous detection of topography and lateral forces. The AFM was used in the contact mode both for lithography and for imaging. Contact-mode V-shaped silicon nitride cantilevers with pyramidal tips, and with force constants between 0.03 N/m and 0.1 N/m, were used. Within each experiment, the same AFM cantilever tip was used both for nanolithography and for subsequent AFM imaging. The position of the tip was controlled by a lithography mode of our software, which at the same time allows control of the electrochemical potential. All AFM images were taken in situ under the electrolyte within the electrochemical cell. All images represent original raw data without filtering or image processing.

**Electrochemical cell and controller:** The experiments were performed in an electrochemical cell approximately 20 mm in diameter, with Cu reference and counter electrodes (copper wires of 0.5 mm diameter, Goodfellow) and a glass substrate with an evaporated gold film as the working electrode. The electrochemistry was controlled by a home-built, low-noise potentiostat, which was controlled by a computer. Cyclic voltammograms were measured both before and after each experiment. Aqueous solutions of 50 mM H_2_SO_4_ (Suprapur, Merck) with 1 mM CuSO_4_ (p.a., Merck) were used as electrolytes. The potentials given in this article were measured against Cu/Cu^2+^ electrodes. Electrochemical deposition was performed at an overpotential of −60 mV. This potential was applied while the surface was locally mechanically depassivated with the AFM tip, resulting in the site-selective, local copper deposition.

**Sample preparation:** Glass slides of approximately 20 mm in diameter were used as samples, and were rinsed and sonicated, first in acetone and subsequently in ethanol. Prior to evaporation of a 50 nm gold film, a 3–4 nm Cr film was evaporated as an adhesion layer. The layer thickness was measured in situ in the vacuum chamber during evaporation by means of a quartz microbalance, and the base pressure in the vacuum chamber during evaporation was in the range of 10^−6^ mbar.
